# CHA2DS2-VASc Score Predict No-Reflow Phenomenon in Primary Percutaneous Coronary Intervention in Primary Percutaneous Coronary Intervention

**DOI:** 10.15171/jcvtr.2018.08

**Published:** 2018-03-18

**Authors:** Fardin Mirbolouk, Mahboobeh Gholipour, Arsalan Salari, Maryam Shakiba, Jalal Kheyrkhah, Vahid Nikseresht, Nozar Sotoudeh, Negar Moghadam, Mohammad Jaafar Mirbolouk, Mani Moayeri far

**Affiliations:** ^1^Cardiovascular Diseases Research Center, Department of Cardiology, Heshmat Hospital, School of Medicine, Guilan University of Medical Sciences, Rasht, Iran; ^2^Cardiovascular diseases research Center, Guilan University of Medical Sciences, Rasht, Iran; ^3^Healthy Heart Research Center, Guilan University of Medical Sciences, Rasht, Iran; ^4^Cardiovascular diseases research Center, Department of cardiology, Guilan University of Medical Sciences, Rasht, Iran

**Keywords:** CHA2DS2-VASc Score, STEMI, Primary PCI, No-reflow

## Abstract

***Introduction:*** No-reflow is one of the major complications of primary PCI in patients with acute ST elevation myocardial infarction. This phenomenon is associated with adverse outcomes in these patients. In the current study, we evaluated the effectiveness of CHA2DS2-VASc score in predicting no-reflow phenomenon. CHA2DS2-VASc score is a risk stratification method to estimate the risk of thromboembolism in patients with atrial fibrillation.

***Methods: *** In total, 396 patients with ST elevation myocardial infarction who had undergone primary PCI were evaluated in our study. Based on post interventional TIMI flow rate results, the patients were divided into two groups: control group (294 patients) and no-reflow group (102 patients). The CHA2DS2-VASc score was calculated for each participant. Multivariate regression analysis was performed to determine the predictive value of this score.

***Results: ***Our findings showed that CHA2DS2-VASc score can predict no-reflow independently (odds ratio: 3.06, 95%, confidence interval: 2.23-4.21, P <0 .001). Moreover, lower systolic blood pressure, higher diastolic blood pressure, grade 0 initial TIMI flow rate and smaller stent size were other independent predictors of the no-reflow in our study. We also defined a cut off value of ≥ 2 for the CHA2DS2-VASc score in predicting the no-reflow with a sensitivity of 88% and specificity of 67%, area under curve: 0.83 with 95% CI (0.79-0.88).

***Conclusion: *** The CHA2DS2-VASc score could be used as a simple applicable tool in the prediction of no-reflow before primary PCI in the acute ST elevation myocardial infarction patients.

## Introduction

In patients with ST-segment elevation myocardial infarction (STEMI), the purpose of primary percutaneous coronary intervention (PCI) is immediate return of normal blood flow in the infarct-related artery.^1,2^ Nevertheless, no-reflow phenomenon is a major challenging disadvantage of this procedure. No-reflow is defined as inadequate myocardial perfusion despite mechanical reopening of the culprit lesion with PCI. This phenomenon is related to higher incidence of complications, and short- and long-term morbidity and mortality in acute STEMI patients.^3,4^

This phenomenon occurs in 0.6% to 5% of elective PCIs, but a higher incidence has been reported in patients who underwent primary PCI.^5,6^ A multifactorial and complex pathophysiology has been suggested for mechanism of this event.^4,7,8^ Unfortunately, there is no widely accepted risk stratification method for the prediction of this complication.

CHA2DS2-VASc score is a clinical predictor of thromboembolism events and is recommended in clinical guidelines for oral anticoagulant therapy in patients with nonvalvular atrial fibrillation.^9^ The components of this score are related to atherosclerosis, vascular spasm and microvascular dysfunction similar to common risk factors of the no-reflow.^10^

In this study, we evaluated the CHA2DS2-VASc score as a simple tool for predicting the no-reflow among patients with STEMI who underwent primary PCI.

## Material and Methods

### Study population

This retrospective analytic-cross sectional study used the data of 396 consecutive patients from October 2015 to October 2016 who were admitted to our cardiovascular center with a diagnosis of acute STEMI and underwent primary PCI Acute STEMI was diagnosed when patients had symptoms of acute myocardial infarction and new ST segment elevation in at least 2 contiguous leads of ≥0.2 mV in men or ≥0.15 mV in women in leads V2 to V3 and/or of ≥1 mm (0.1 mV) in other contiguous leads or new left bundle branch block, later confirmed by creatine kinase (CK) and CK-myocardial band (CK-MB) isoenzyme increases and/or troponin increases.^11^ Patients with symptoms lasting more than 12 hours before admission, no intervention because of patent or normal coronary arteries, stenosis in the venous graft as culprit lesion, decision of emergency surgery because of inappropriate coronary anatomy for intervention and coronary artery dissection as a procedural complication were excluded from the study. Bedside 12-lead electrocardiography and routine blood tests were obtained from all admitted patients. Bedside echocardiography was also performed for the patients. All data were acquired from hospital records retrospectively.

### Coronary angiography and primary PCI

All the patients who were candidate for primary PCI, received 325 mg of aspirin and a single loading dose of 600 mg clopidogrel at the time of diagnosis of STEMI coronary angiography was performed using standard technique. Immediately after the decision of coronary intervention, 50-70 unit/kg of intravenous bolus dose of unfractionated heparin was administered to the patients who were not treated with enoxaparin before the coronary angiography. For patients who have received an initial enoxaparin dose of 1 mg/kg before the angiography, no additional booster dose of enoxaparin was administered within 8 hours of the first dose. An additional booster enoxaparin of 0.3 mg/kg was given intravenously between 8–12 hours after the first dose. Thrombus aspiration catheter usage and administration of eptifibatide (a glycoprotein IIb/IIIa Inhibitors with a 180 mcg/kg IV bolus dose over 1-2 minutes, then continuous infusion 2 mcg/kg/min with another 180 mcg/kg IV bolus dose 10 minutes after first one for at least 12 hours) were chosen according to the interventional cardiologist’s decision. The TIMI flow grades were evaluated by 2 blind cardiologists. The frame rate of cine images were 30‏ frames per seconds. Analysis of cineangiograms was performed by using an Axiom (Siemens Medical Solution, Erlangen, Germany) workstation.

### Definitions

The study population was divided into two groups of control and no-flow according to their final angiographic TIMI flow rates resulting from primary PCI. The control group was defined as the TIMI flow rate >2 and the no-reflow group was defined as the TIMI flow rate ≤2, despite mechanical reopening of the infarct-related artery in patients without dissection of the coronary artery.^12^ Definition of the TIMI flow grades was as follows: Grade 0 refers to no flow at all after the culprit lesion. In grade 1, the contrast material flow after occlusion site but fails to opacify the entire artery. Grade 2 refers to opacification of the entire artery distal to the obstruction point, however the flow is slower than normal, and grade 3 refers to normal coronary flow.^13^

The CHA2DS2-VASc score was the sum of 1 point each for the presence of congestive heart failure, hypertension, diabetes mellitus, age of 65 to 74‏ years, female sex, and vascular diseases (history of MI, peripheral arterial disease, or complex aortic plaques) and 2 points for age ≥ 75 years and a history of stroke or transient ischemic attack (TIA).^14^ Congestive heart failure, hypertension, diabetes mellitus and hyperlipidemia was diagnosed based on patient’s past medical history.

Peripheral arterial disease (PAD) was defined as the documentation of stenosis of 50% in noncoronary arteries. Definition of the chronic renal failure was based on a creatinine clearance of less than 60 mL/minute, which was calculated by Cockroft formula.^15^

### Statistics

Quantitative variables were defined as mean value ± standard deviation (SD), and qualitative variables were defined as frequency and percentage.

The Kolmogorov-Smirnov test was used to evaluate whether the distribution of continuous variables was normal. Categorical and continues variables, were analyzed using chi-square test and independent sample *t* test, respectively. Multivariate logistic regression analyses were performed to determine the independent predictors. Variables that could be a predictor of no-reflow with a significant *P* value were entered into multivariate analysis. The results of univariate and multivariate regression analyses were presented as odds ratio with 95% CI. The ROC curve was also used to demonstrate the sensitivity and specificity of CHA2DS2-VASc score and its cut-off value in predicting the no-reflow. A P value < 0.05 was considered as statistically significant. Statistical analyses were conducted using STATA version 13.0.

## Results

Demographics, clinical and angiographic data of the patients are listed in Table 1. The study population consisted of 396 patients (mean age 58 ± 11 years, 104 women [26%]), of whom 294 patients were in the control group and 102 patients were in the no-reflow group.

**Table 1:** Demographic, clinical, and angiographic characteristic of the patients

**Variables**	**Control, n = 294**	**No-reflow, n = 102**	***P*** ** Value**
Age, years, mean (SD)	57 (11)	63 (11)	<0.001
Female gender, n (%)	66 (22.4)	38 (37.2)	0.003
History of heart failure, n (%)	4 (1.3)	23 (22.5)	<0.001
Hypertension, n (%)	92 (31.3)	74 (72)	<0.001
Diabetes mellitus, n (%)	53 (18)	51 (51)	<0.001
Hyperlipidemia, n (%)	64 (21.7)	50 (49)	<0.001
History of stroke/TIA, n (%)	4 (1.3)	6 (5.9)	0.01
Vascular disease, n (%)	29 (9.8)	46 (45.1)	<0.001
Previous MI, n (%)	26 (8.8)	44 (43.1)	<0.001
Previous by-pass surgery, n (%)	5 (1.7)	9 (2.3)	0.19
Peripheral arterial disease, n (%)	1 (0.3)	5 (4.9)	0.001
Smoking, n (%)	119 (40.5)	27 (26.5)	0.01
CHA2DS2-VASc score, mean (SD)	1.1 (1.1)	3 (1.4)	<0.001
Anemia, n (%)	73 (24.8)	43 (42.1)	0.001
Serum creatinine, mean, mg/dl(SD)	1 (0.2)	1.13 (0.55)	0.002
GFR, ml/min/1.73 m², mean (SD)	79 (14.9)	69 (17.7)	<0.001
Chronic renal failure, n (%)	25 (8.5)	25 (24.5)	<0.001
LV ejection fraction, (%), mean (SD)	38.2 (7.9)	34 (8.5)	<0.001
Systolic BP, mm Hg, mean (SD)	136.5 (23.4)	126.2 (29.6)	<0.001
Diastolic BP, mm Hg, mean (SD)	80.7 (12.6)	75.9 (16)	0.002
MI type, n (%)			
Anterior	165 (56.2)	65 (63.7)	0.18
Non anterior‏	129 (43.8)	37 (36.2)	
Initial TIMI flow rates, n (%)			
TIMI = 0	198 (67.3)	97 (95.1)	<0.001
TIMI ≥ 1 (1,2,3)	96 (32.6)	5 (4.9)	
Lesion length, mm, mean (SD)	17.7 (7.7)	16.9 (7)	0.39
Stent length, mm, mean (SD)	27(7.5)	26(7)	0.57
Stent diameter, mm, mean(SD)	3 (0.29)	2.95 (0.26)	0.009
Eptifibatide infusion, n (%)	189 (64.2)	73 (71.5)	0.18
Thrombus aspiration, n (%)	96 (32.6)	38 (37.2)	0.39
Time to PCI, minute, mean(SD)	160.5 (128.6)	181.3 (123.6)	0.15
In-hospital mortality, n (%)	3 (1)	6 (5.9)	0.005

Abbreviations: TIA, transient ischemic attack; MI, myocardial infarction; GFR, glomerular filtration rate; LV, left ventricle; BP, blood pressure; IRQ, interquartile range; LAD, left anterior descending; LCX, left circumflex; RCA, right coronary artery; PCI, percutaneous coronary intervention.

Compared to control group, patients in the no-reflow group were older (63 ± 11 vs 57 ± 11, *P* < 0.001) and prevalence of grade 0 initial TIMI flow rates was significantly higher in them (95.1% versus 67.3%, *P* < 0.001).

The mean CHA2DS2-VASc score was 1.6 ± 1.4 and it was significantly higher in the no-reflow group compared to the control group (3 ± 1.4 versus 1.1 ± 1.1, *P* < 0.001). Furthermore, in comparison to control group, all components of CHA2DS2-VASc score, including history of heart failure, hypertension, age between 65 and 74, diabetes mellitus, history of stroke/transient ischemic attack, vascular disease, age ≥75, and female gender were significantly higher in the no-reflow group. History of previous MI and peripheral arterial disease were more common in the no-reflow group, but history of previous by-pass surgery did not differ between the two groups (2.3% versus 1.7%, *P* = 0.19).

Patients with the no-reflow had significantly lower mean glomerular filtration rate, left ventricle ejection fraction, systolic blood pressure (SBP) and diastolic blood pressure (DBP) and they had significantly higher in-hospital mortality rate (5.9% versus 1%, *P* = 0.005) compared to the control group. Anemia, chronic renal failure and hyperlipidemia were also more prevalent in them than in the control group.

There was no significant difference between two cohorts in duration from symptoms initiation to primary PCI (181.3 ± 123.6 minutes versus 160.5 ± 128.6 minutes, *P* = 0.15).

Regarding the angiographic findings, lower stent diameter was related to no-reflow, but stent length and lesion length did not differ between the two groups.

Use of eptifibatide infusion (71.5% versus 64.2%, *P* = 0.18) and thrombus aspiration (37.2% versus 32.6%, *P* = 0.39) based on operator decision were similar in the two cohorts. These variables interpreted as a consequence of high risk lesions and therefore were not entered in the regression analysis.

Variables that had significant *P* value in descriptive analysis were entered into univariate and multivariate regression analysis to determine potential risk factors of no-reflow. Results of this analysis are illustrated in Table 2. Individual components of CHA2DS2-VASc score as a risk factor of the no-reflow were not entered in this analysis to avoid multicollinearity.

**Table 2:** Univariate and multivariate regression analysis of predictors of no-reflow

**Variables**	**Unadjusted OR (95% CI)**	***P*** ** Value**	**Adjusted OR (95% CI)**	***P*** ** Value**
CHA2DS2-VASc 1-SD increase	2.75 (2.21-3.43)	<0.001	3.06 (2.23-4.21)	<0.001
Hyperlipidemia	3.45 (2.14-5.56)	<0.001	1.62 (0.84-3.12)	0.14
Anemia	2.20 (1.37-3.54)	0.001	1.33 (0.67-2.65)	0.40
CRF	3.49 (1.89-6.42)	1.89	0.74 (0.21-2.59)	0.64
GFR 1-SD increase	0.54 (0.42-0.69)	<0.001	1.15 (0.68-1.94)	0.57
Serum creatinine 1-SD increase	1.55 (1.11-2.15)	0.009	1.02 (0.69-1.52)	0.88
Smoking	0.52 (1.37-3.54)	0.012	1.85 (0.88-3.90)	0.10
SBP 1-SD increase	0.65 (0.51-0.83)	0.001	0.45 (0.26-0.76)	0.003
DBP 1-SD increase	0.68 (0.53-0.87)	0.53	1.91 (1.11-3.27)	0.018
LVEF 1-SD increase	0.59 (0.47-0.75)	<0.001	0.79 (0.57-1.10)	0.17
Initial TIMI flow rate ≥1	0.48 (0.38-0.62)	<0.001	0.06 (0.02-0.20)	<0.001
Stent diameter, 1-SD increase	0.63 (0.51-0.78)	<0.001	0.70 (0.52-0.95)	0.023

Abbreviations: SD, standard deviation; CRF, chronic renal failure; GFR, glomerular filtration rate; SBP, systolic blood pressure ; DBP, diastolic blood pressure; LVEF, left ventricular ejection fraction.

Results from the multivariate logistic regression analysis showed that CHA2DS2-VASc score is a significant independent predictor (odds ratio [OR]: 3.06, 95% CI: 2.23-4.21, *P* < 0.001) of the no-reflow. Moreover, other independent predictors of the no-reflow in our study were lower SBP, higher DBP, grade 0 initial TIMI flow rate and lower stent diameter.

**Figure 1:**
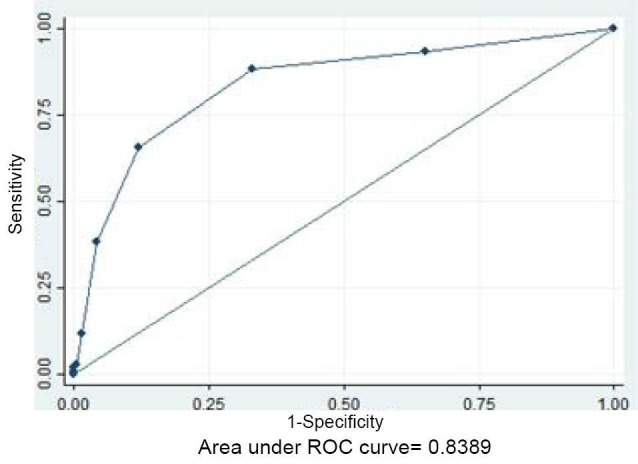
ROC curve of CHA2DS2-VASc score =2 in predicting no-reflow.

Predictive power of individual characteristics of the CHA2DS2-VASc score was determined in a separate univariate and multivariate regression analysis and is shown in Table 3. In multivariate analysis of the CHA2DS2-VASc score components, congestive heart failure, hypertension, age 65 to 74, age ≥75, diabetes mellitus and vascular disease predict the no-reflow independently with a higher odds ratio for the congestive heart failure (OR: 9.76, CI: 2.81-33.81, *P* < 0.001). Then, we performed a ROC analysis as depicted in Figure 1 for evaluating cutoff value of CHA2DS2-VASc score in predicting the no-reflow. Our study showed that CHA2DS2-VASc score ≥2 can be used as a predictor of the no-reflow in patients presented with acute ST elevation myocardial infarction with a sensitivity of 88% and specificity of 67%, area under curve: 0.83 with 95% CI (0.79-0.88).

**Table 3:** Univariate and multivariate analysis of predictive power of individual components in CHA2DS2-VASc score for no-reflow

**Variables**	**Unadjusted OR (95% CI)**	***P*** ** Value**	**Adjusted OR (95% CI)**	***P*** ** Value**
Congestive heart failure	21.10 (7.09-62.81)	<0.001	9.76 (2.81-33.81)	<0.001
Hypertension	5.80 (3.52-9.56)	<0.001	4.09 (2.28-7.35)	<0.001
Age ≥75	2.03 (1.01-4.06)	0.045	3.26 (1.35-7.87)	0.008
Age 65-74	2.50 (1.43-4.37)	0.001	2.19 (1.04-4.63)	0.03
Diabetes mellitus	4.54 (2.78-7.41)	<0.001	3.44 (1.87-6.31)	<0.001
Stroke, TIA	4.53 (1.25-16.39)	0.021	2.16 (0.43-10.66)	0.34
Vascular disease	7.50 (4.34-12.96)	<0.001	3.73 (1.86-7.47)	<0.001
Female gender	2.05 (1.26-3.33)	0.004	1.54 (0.83-2.85)	0.16

Abbreviations: CI, confidence interval; TIA, transient ischemic attack.

## Discussion

Our study declares the usefulness of CHA2DS2–VASc score in predicting no-reflow phenomenon after primary PCI in STEMI patients. Moreover, we reached a cut-off value of ≥2 for predicting no-reflow possibility in these patients. These findings are in concordance with a previous study of Ipek et al^16^ which evaluated the predictive power of CHA2DS2-VASc score in 1781 patients with STEMI who underwent primary PCI.

Primary PCI is the preferred revascularization method in most patients with a diagnosis of acute STEMI, but acute reduction in myocardial blood flow after this procedure despite a patent epicardial coronary artery, the so-called “no-reflow phenomenon”, leads to adverse outcomes in these patients.^3,4,17^

Although there are few experimental therapies to solve this complication but no standard treatment has yet been defined.^1,4,18,19^ The reason is complexity of proposed pathophysiologic mechanisms for no-reflow phenomenon.

Some studies suggest deferring stent strategy to reduce no-reflow after PCI.^20^ Using a simple and quick scoring system for risk stratification of no-reflow in STEMI patients who are candidate for primary PCI, enables physician to choose the best treatment strategy. In this regard due to similarity of underlying mechanisms of no-reflow phenomenon with major risk factors of thromboembolic events among patients with atrial fibrillation we chose CHA2DS2-VASc score for deciding about revascularization method.

Congestive heart failure,^16,21^ hypertension and ischemic cardiomyopathy,^5^ as well as age 65-74 years and age ≥75^16^ were predictors of no-reflow in our study similar to previous studies.

In our cohort, multivariate analysis showed that diabetes mellitus and peripheral arterial disease are associated with no-reflow during primary PCI. This finding is not similar to earlier studies which did not find diabetes mellitus as a predictor in spite of demonstrating an association between hyperglycemia and no-reflow.^22^ Similarly, although peripheral arterial disease can increase mortality and morbidity in ACS patients but no specific study has shown the correlation between PAD and no-reflow.^23,24^ Indeed impaired microvascular reperfusion due to diabetes explains this association and in the same manner the similar vascular mechanism of PAD with coronary artery disease can determine the association between PAD and no-reflow.

Various clinical and angiographic predictors of no-reflow have been proposed in previous studies. For example, thrombus burden and its angiographic features,^25^ lower stent diameter and length lesion >20 mm^20,26^ are independent predictors of no-reflow. Our results also showed that lower stent diameter can predict no-reflow. Based on our findings grade 0 TIMI flow rate at initial angiography also was an independent predictor of no-reflow similar to another previous study.^27^

Our findings also revealed that lower systolic blood pressure is correlated with increased risk of no-reflow independently. It might be related to reduction of coronary arterial perfusion pressure due to decreased blood pressure. Moreover, swelled myocardial cells concomitant with interstitial edema might lead to microvascular compression.^28^ This mechanism and oxidative stress of ischemic endothelial cells along with vasoconstriction can reduce perfusion of microvasculator and lead to no-reflow.

Many of the risk factors such as hypertension, diabetes mellitus and female gender that were discussed above are associated with microvascular dysfunction.^29,30^ There is also an association between abnormal vascular function and stroke.^31^ Although, in our cohort after multivariate analysis there was no significant relationship between no-reflow and female gender and stroke.

CHA2DS2-VASc score used to predict thromboembolic events in patients with atrial fibrillation rhythm,^9^ consisted of similar risk factors of microvascular dysfunction as an important mediator of no-reflow. The components of this score are common risk factors of atherosclerosis, vascular spasm, microvascular dysfunction as well as no-reflow and stroke.^10^ Thus, it is anticipated that CHA2DS2-VASc score could predict no-reflow phenomenon as we confirmed in our cohort. Furthermore, use of this score is very simple and makes it a quick tool to predict no-reflow before primary PCI.

### Study Limitations

Retrospective design of our cohort, is one of the limitations of the study. Many of variables in this study were based on a review of pervious clinical history of the patients in an acute phase of STEMI and it may affect our results. Another important issue is limited sample size. Finally, there are multiple risk factors for the no-reflow that we did not assess them and it might have affected our multivariate analysis.

In conclusion, we showed the CHA2DS2-VASc score potential to predict the no-reflow phenomenon in the patients with STEMI before primary PCI and this finding was similar to a previous study.^16^ Although, it is suggested that the predictive power of this score should be reevaluated in a prospective study with a larger sample size and with more comprehensive risk factors to confirm our results.

## Ethical approval

Our study was approved by the local ethics committee. (Ethical No. IR.GUMS.RES.1395.30). All the participants filled out the signed consent form.

## Competing interests

All authors declare no competing financial interests exist.
